# Mutation spectrum of *PRPF31*, genotype-phenotype correlation in retinitis pigmentosa, and opportunities for therapy

**DOI:** 10.1016/j.exer.2020.107950

**Published:** 2020-03

**Authors:** Gabrielle Wheway, Andrew Douglas, Diana Baralle, Elsa Guillot

**Affiliations:** a Faculty of Medicine, University of Southampton, Human Development and Health, UK; b University Hospital Southampton NHS Foundation Trust, UK; c Department of Engineering, Design and Mathematics, University of the West of England, Bristol, UK

## Abstract

Pathogenic variants in pre-messenger RNA (pre-mRNA) splicing factor 31, *PRPF31*, are the second most common genetic cause of autosomal dominant retinitis pigmentosa (adRP) in most populations. This remains a completely untreatable and incurable form of blindness, and it can be difficult to predict the clinical course of disease. In order to design appropriate targeted therapies, a thorough understanding of the genetics and molecular mechanism of this disease is required. Here, we present the structure of the *PRPF31* gene and PRPF31 protein, current understanding of PRPF31 protein function and the full spectrum of all reported clinically relevant variants in *PRPF31*. We delineate the correlation between specific *PRPF31* genotype and RP phenotype, suggesting that, except in cases of complete gene deletion or large-scale deletions, dominant negative effects contribute to phenotype as well as haploinsufficiency. This has important impacts on design of targeted therapies, particularly the feasibility of gene augmentation as a broad approach for treatment of *PRPF31*-associated RP. We discuss other opportunities for therapy, including antisense oligonucleotide therapy and gene-independent approaches and offer future perspectives on treatment of this form of RP.

## Highlights

• *PRPF31* is the second most common cause of autosomal dominant retinitis pigmentosa and a potential target for gene therapy.
• We present all reported pathogenic variants in *PRPF31* as a resource for clinicians, diagnostic genetics labs, and researchers.
• Genotype-phenotype correlations suggest that, dominant negative effects contribute to disease in addition to haploinsufficiency.
• This finding has important impacts on the suitability of gene augmentation approaches across all mutation types.
• This finding may aid prognosis of disease in *PRPF31*-associated RP patients.

## 1 Introduction

### 1.1 Pre-mRNA splicing

Human pre-mRNA splicing factor 31 (PRPF31) is a component of the spliceosome, the huge macromolecular ribonucleoprotein (RNP) complex which catalyses the splicing of pre-messenger RNAs (pre-mRNAs) to remove introns and produce mature mRNAs(Will and Luhrmann, 2011).

Pre-mRNA splicing is a core function in all eukaryotic cells. The vast majority of genes have multiple exons and introns, and around 95% of these multiexon genes undergo alternative splicing(Pan et al., 2008). Alternative splicing allows increased organism complexity without increasing genome size, and helps to explain the c-value paradox; the observation that phenotypic complexity in the eukaryotic domain is not proportional to genome size.

**Fig. 1:**
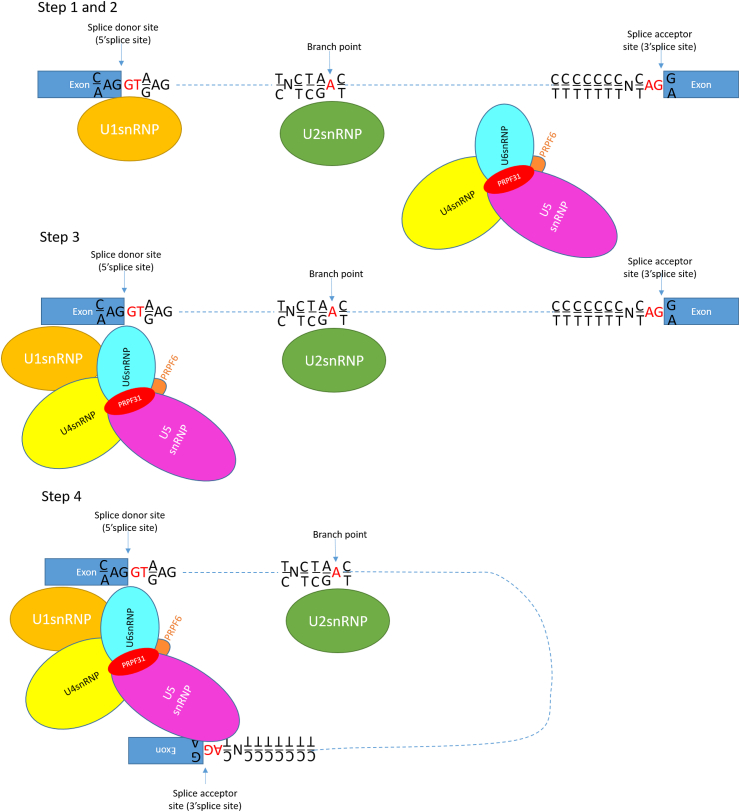
**Schematic representation of the first four steps of pre-mRNA splicing by the major spliceosome, with PRPF31 shown in red.** In step 1, U1snRNP recognises and binds the splice donor site (the 5′ splice site). In step 2, binding of U1snRNP to the splice donor site promotes the binding of U2snRNP to the branch site. Independently of this, the U4/U6.U5 tri-snRNP forms in the cell. In step 3, the U4/U6.U5 tri-snRNP is recruited to the pre-mRNA, where U6snRNP replaces U1snRNP. This forms the catalytically active spliceosome, which in step 4 cuts away the intron and joins the exons through two transesterification reactions. (For interpretation of the references to colour in this figure legend, the reader is referred to the Web version of this article.)

The spliceosome is composed of 5 small nuclear RNAs (snRNAs), U1–U5, and many proteins, together making 5 snRNPs. In the process of splicing, U1snRNP recognises and binds the splice donor site (the 5’ splice site), and promotes the binding of U2snRNP to the branch site. Independently of this, the U4/U6.U5 tri-snRNP forms in the cell, and is recruited to the pre-mRNA, where U6snRNP replaces U1snRNP. This forms the catalytically active spliceosome, which cuts away the intron and joins the exons through two transesterification reactions (Fig. 1).

### 1.2 PRPF31, splicing and retinal disease

The *S. cerevisiae* yeast homologue of PRPF31, Prp31, was cloned and identified as a key splicing factor in 1996 (Weidenhammer et al., 1996), and later was shown to be essential for the association of the U4/U6.U5 tri-snRNP with pre-spliceosomes(Weidenhammer et al., 1997). It was subsequently found to play a role in both splicing and meiosis in *S. pombe*(Bishop et al., 2000). Unexpectedly, in 2001, it was discovered that heterozygous pathogenic variants in *PRPF31* are associated with retinitis pigmentosa (RP), an inherited retinal dystrophy affecting 1:2000 to 1:3500 people worldwide(Vithana et al., 2001). This was surprising because pre-mRNA splicing factors are highly conserved from yeast to man with a core function in all cells. Intuitively, it would be expected that a defect in a core spliceosomal protein should have an impact on all cells, not just retinal cells.

The original paper described seven different pathogenic variants in four families and three simplex cases. These included mutations in the region of the splice site, leading to inactivation of a splice acceptor site, inactivation of a splice donor site, two missense changes, three frameshift variants and an in-frame duplication(Vithana et al., 2001).

Since then, and particularly since the advent of massively parallel sequencing technologies, it has become clear that pathogenic variants in *PRPF31* are a major cause of autosomal dominant RP (adRP). Indeed they are the second most common genetic cause of adRP in most populations, accounting for 6% of US cases (Sullivan et al., 2013), 8% of Spanish, French and French-Canadian cases (Martin-Merida et al., 2018; Audo et al., 2010; Coussa et al., 2015), 8.9% of cases in North America (Daiger et al., 2014), 10–11.1% of Chinese cases (Lim et al., 2009; Xu et al., 2012) and 10.5% of Belgian cases(Van Cauwenbergh et al., 2017).

However, this is likely to be an underestimate due to non-penetrance of this form of RP (Rose and Bhattacharya, 2016). It is common to see very variable severity of eye disease in different members of the same family with the same pathogenic *PRPF31* variant. Furthermore, obligate carriers may be totally asymptomatic, showing complete non-penetrance. This complicates attempts to co-segregate *PRPF31* variants with clinical disease and makes genetic diagnosis difficult, likely contributing to an underestimation of the prevalence of RP associated with *PRPF31* variants.

The genetic mechanism controlling incomplete penetrance remains unclear, but a fairly consistent observation of correlation between expression level of the non-mutant copy of *PRPF31* and disease severity has been reported.(Rio Frio et al. 2008b, 2009; Rivolta et al., 2006).

This varied expression can be explained by a number of factors including:

- expression quantitative trait loci (eQTLs) (on ch.14q21-23) in trans with *PRPF31(*Rio Frio et al., 2008a)
- variable level of expression of *CNOT3*, a trans-acting epistatic factor which is genetically linked to *PRPF31* and regulates expression of *PRPF31. CNOT3* encodes a subunit of the Ccr4-not transcription complex, which binds to the promoter of *PRPF31* and represses transcription of *PRPF31.* An intronic variant in *CNOT3* determines its level of expression and thus how efficiently *PRPF31* expression is downregulated. The alleles of *CNOT3* inherited determine the expression of non-mutant PRPF31 and thus whether a person will be affected by the disease(Venturini et al., 2012; Rose et al., 2014).
- the number of minisatellite repeat elements (MSR1) adjacent to the *PRPF31* core promoter, which determines the level of transcriptional repression of the non-mutant PRPF31.4 MSR1 copies are associated with higher non-mutant PRPF31 expression and are found in non-symptomatic carriers only(Rose et al., 2016).

On the basis of these observations, the mechanism of incomplete penetrance in this form of RP has been described as ‘variant haploinsufficiency’, in which the absence of a second wild-type PRPF31 allele is sometimes sufficient to produce disease, and sometimes is not, depending on the nature of the mutant allele inherited *and* the nature of the wild-type allele inherited. So the severity of the resultant disease depends on both the type of mutant allele inherited (ie complete loss-of-function, gain-of-function or hypomorphic), the level at which this allele is expressed, and the level at which the wild-type allele is expressed (Rose and Bhattacharya, 2016). This form of variant haploinsufficiency has only been described in a very few Mendelian disorders, making the mechanism of variable penetrance in this disease quite unique (Rose and Bhattacharya, 2016).

### 1.3 *PRPF31* gene and PRPF31 protein structure

*PRPF31* is a 16.3 kb gene on chromosome 19 which encodes 9 different transcripts, 6 of which are protein coding. The largest, most widely expressed transcript consists of 14 exons; 1 non-coding and 13 coding, which produces a 499 amino acid protein of 55 kDa in size, pre-mRNA splicing factor 31, PRPF31.

**Fig. 2:**
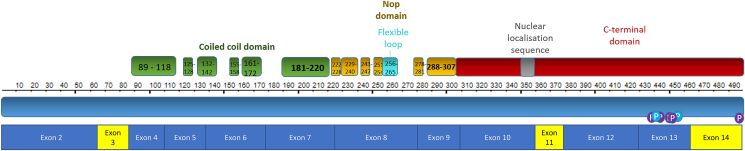
Schematic representation of the protein and cDNA structure of PRPF31, showing major structural domains encoded by each exon.

PRPF31 contains several important functional domains; the flexible loop, Nop domain, coiled-coil domain and tip. Recent advances in spectroscopy and microscopy methods such as NMR and cryo-electron microscopy have allowed accurate resolution of the crystal structure of proteins of the spliceosome, including PRPF31, in their native conformations at different points during splicing(Agafonov et al., 2016; Bertram et al. 2017a, 2017b; Haselbach et al., 2018). These studies have revealed that PRPF31 contains a conserved Nop domain (residues 222–254 and 278–307), with regions for binding protein and RNA(Liu et al., 2007). This Nop domain has relaxed sequence conservation in PRPF31, but it retains high specificity for binding U4 or U4atac and 15.5K protein (Liu et al., 2007). The flexible loop (residues 256–265) protects the exposed C4’ atoms of residues 37 and 38 from attack by free radicals, to protect the RNA without directly contacting it(Liu et al., 2007). The protein also has several phosphorylation sites, clustered in the C-terminus(Liu et al., 2007). PRPF31 contains a nuclear localisation sequence, NLS, which allows it to be targeted to the nucleus after translation (Fig. 2).

### 1.4 PRPF31 protein function

PRPF31 is required for tri-snRNP assembly in human cells(Makarova et al., 2002). With PRFP6, PRPF31 forms an essential connection between the U4/U6 and U5 snRNPs. siRNA knockdown of PRPF31 results in inhibition of tri-snRNP formation and nuclear accumulation of U5 mono-snRNPs and U4/U6 di-snRNPs containing U4/U6 proteins and the U4/U6 recycling factor p110(Schaffert et al., 2004).

The specific function of PRPF31 in retinal cells remains less clear. It remains unclear whether the photoreceptor cells are the primary affected cells in RP associated with PRPF31, with a number of studies suggesting that the RPE is the primary affected tissue(Farkas et al., 2014; Hamieh and Nandrot, 2019; Valdés-Sánchez et al., 2019). Retinal cells are highly metabolically active, with a high demand for ATP and protein anabolism as around 10% of protein from photoreceptor outer segments is shed every day. Rates of metabolism in photoreceptors are similar to dividing tumour cells, and undergo extensive anaerobic glycolysis rather than oxidative phosphorylation to produce energy, in what is termed the ‘Warburg effect’(Ng et al., 2015; Rajala et al., 2016). The reliance on glycolysis seems to promote efficient protein anabolism in photoreceptors(Chinchore et al., 2017). However, the photoreceptors still require mitochondria to produce a proportion of their ATP via oxidative phosphorylation(Grenell et al., 2019). It has been postulated that photoreceptor cells have a greater demand for pre-mRNA splicing factors to meet this metabolic demand, but evidence to support this hypothesis is inconsistent. Some studies have reported higher levels of PRPF31 expression in retina than in other tissues (Cao et al., 2011) but other studies show a consistent level of expression in all tissues, with no significantly higher expression in retina or any other tissue(Yuan et al., 2005).

Related to this elevated rate of oxidative phosphorylation, retinal cells are subject to much higher rates of oxidative damage, including UV-induced photooxidative damage, which may explain the retinal-specific phenotype of RP associated with pre-mRNA splicing factor mutations.(Comitato et al., 2007; Shinde et al., 2016; Jin et al., 2011; Schmidt-Kastner et al., 2008). In patients expressing mutant forms of pre-mRNA splicing factors, it has been shown that proteins have reduced solubility, which can lead to formation of protein aggregates, and it has been suggested that the environment of UV-induced photooxidative damage in the photoreceptors makes these cells specifically prone to degeneration(Wheway et al., 2019; Valdés-Sánchez et al., 2019; Wilkie et al., 2006; Yin et al., 2011; Bryant et al., 2019). This splicing-independent disease mechanism is appealing because there is inconsistent evidence of splicing defects in cells carrying PRPF31 mutations. Studies seem to suggest that expression of mutant *PRPF31* affects splicing of some transcripts but not others.

Immunoprecipitation of splicing complexes from *PRPF31* mutant retinal cells showed that mutant PRPF31 proteins significantly inhibited pre-mRNA splicing of intron 3 in the rhodopsin (RHO) gene(Yuan et al., 2005). In primary retinal cell cultures, expression of the mutant PRPF31 proteins reduced total RHO expression and caused apoptosis of rhodopsin-positive retinal cells(Alagramam et al., 2001). In a study of patient lymphoblastoid cell lines, splicing efficiency of RPGR intron 9 was significantly decreased in *PRPF31* mutant cell lines but no consistent decrease in the splicing efficiency of U12 and noncanonical U2 introns was seen in *PRPF31* mutant cells(Ivings et al., 2008). In a minigene study, assays using the RHO intron 3 minigene template revealed a direct negative effect on splicing efficiency of mutant PRPF31. However, no effect of the mutation on splicing efficiency could be detected using the longer GNAT1 minigene template or using a full-length RHO transcript, splicing of which had an efficiency of 100%. Similarly, no unspliced RHO transcripts could be detected in RNA from human retina(Wilkie et al., 2008).

Using novel stem cell technologies, recent studies in retinal organoids and retinal pigment epithelium (RPE) derived from induced pluripotent stem cells (iPSCs) from patients with *PRPF31* mutations show decreased efficiency of splicing of E1A minigene(Buskin et al., 2018). RPE from patient iPSCs also show a substantial downregulation of SART1, a U5 snRNP protein important for the formation of the pre-catalytic spliceosome, but no changes in the expression of the U5 protein PRPF8 or the U4/U6 protein PRPF4(Buskin et al., 2018). In both RPE and retinal organoids derived from *PRPF31* patients, the most significantly mis-spliced genes were genes involved in pre-mRNA and alternative mRNA splicing via the spliceosome(Buskin et al., 2018).

Alongside these findings, it was observed that retinal organoids from patients showed differential expression of actin cytoskeleton, ciliary membrane, primary cilium, photoreceptor inner and outer segment, axon terminal and phototransduction proteins. Furthermore, patient organoids showed an enrichment of mis-spliced centriole and microtubule organisation genes. This suggests that centriole and ciliogenesis and cilium function are all regulated by alternative splicing in the retina, and this is defective in patients carrying *PRPF31* mutations(Buskin et al., 2018). These findings were confirmed in independent studies of splicing in PRPF31 siRNA-treated human organotypic retinal cultures(Azizzadeh Pormehr et al., 2019). This is in keeping with earlier studies from ourselves, and others, which showed that siRNA knockdown of pre-mRNA splicing factors including *PRPF3*1 has a specific and significant effect on ciliogenesis(Wheway et al., 2015; Kim et al., 2016). Further investigation showed that these proteins localise to the base of the photoreceptor cilium, classifying these conditions as retinal ciliopathies(Wheway et al., 2015). Recent work developing *PRPF31* gene augmentation therapy has shown rescue of ciliogenesis in *PRPF31* ± RPE cells derived from human patient iPSCs after expression of wild-type PRPF31 delivered by an AAV vector, further suggesting that PRPF31 plays a key role in regulating ciliogenesis in patients(Brydon et al., 2019).

Further work is needed to understand the nature of the splicing factors’ involvement in ciliogenesis and cilium function in the retina, and this work is ongoing. It is possible that PRPF31 and other splicing factors have roles beyond splicing. Many proteins involved in splicing have multiple functions in the cell, such as the proteins of the PRP19 complex which have roles in ubiquitination (Vander Kooi et al., 2006), in DNA damage sensing (Grey et al., 1996; Marechal et al., 2014), DNA damage repair (Zhang et al., 2005), mRNA export (Chanarat et al., 2011) and in mitotic spindle assembly (Hofmann et al., 2013). PRPF31 has been shown to perform splicing-independent functions in mitotic chromosome segregation, although this would not explain disease phenotype in the post-mitotic retina. With deeper understanding of the molecular mechanism of PRPF31 disease arise greater opportunities for developing effective targeted therapies.

### 1.5 *PRPF31* mutation spectrum

**Fig. 3:**
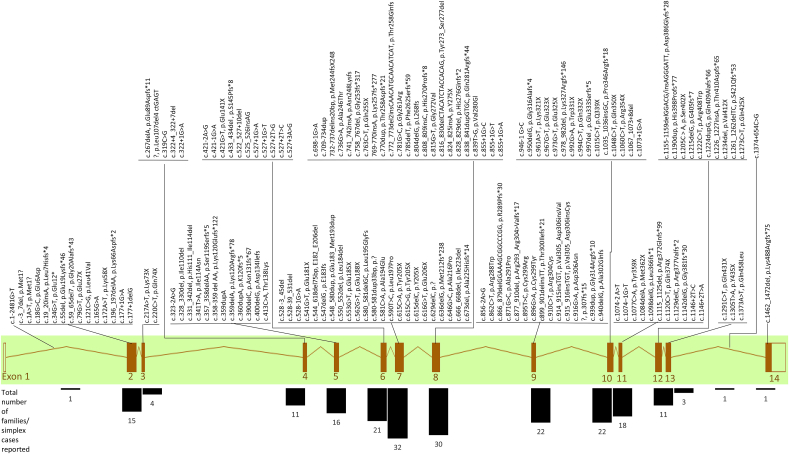
Schematic representation PRPF31 gene, with all reported pathogenic variants labelled above, and total numbers of variants in each intron and exon displayed as a bar chart below. This shows that exons 7 and 8 are most enriched for pathogenic variants.

In order to fully understand the molecular mechanism of RP associated with *PRPF31* variants, it is necessary to fully understand the genetics of this condition. This will aid accurate diagnostics, prognostics and development of targeted therapies. To this end, we have reviewed the literature and the major clinical variant database ClinVar to summarise all reported pathogenic variants in PRPF31 (Table 1). Mutations are spread throughout the gene, but are most common in exons 6–10, particularly exons 7 and 8 (Fig. 3).

**Table 1:** **All reported pathogenic variants in *PRPF31* associated with adRP, from peer-reviewed publications and clinical variant database ClinVar (variants classified as pathogenic only).** The location in cDNA, nature of the variant and impact on protein (if known) is included, alongside age of onset and age at diagnosis, where reported.

exon	cDNA mutation	protein mutation	notes	Original references	Families (n)	Splicing	Frameshift	Nonsense	Missense	Inframe deletion	Inframe duplication/insertion	Indel	Large insertion or deletion	Age of onset	Age at diagnosis
exon 1 (non coding)															
intron 1	c.1-2481G > T		formerly: IVS1+1G > T	Liu et al., 2008 (Liu et al., 2008)	1	1								3	20
exon 2 c.1-177	c.-3_7del	p.Met1?		Sullivan et al., 2013 (Sullivan et al., 2013); Kiser et al., 2019 (Kiser et al., 2019)	2		2							10/17/29	10/58/62
	c.1A > T	p.Met1?		Carss et al., 2017 (Carss et al., 2017)	1										
	c.18G > C	p.Glu6Asp		van Huet et al., 2015 (van Huet et al., 2015)	1				1						
	c.19_20insA	p.Leu7Hisfs*4		Sullivan et al., 2013 (Sullivan et al., 2013)	1		1								
	c.34G > T	p.Glu12*		Van Cauwenbergh et al., 2017 (Van Cauwenbergh et al., 2017)	1		1								
	c.55del	p.Glu19Lysfs*46		Martin-Merida et al., 2018 (Martin-Merida et al., 2018)	1		1								
	c.59_65del7	p.Gly20Alafs*43		Saini et al., 2012 (Saini et al., 2012)	1		1								17
	c.79G > T	p.Glu27X		Waseem et al., 2007 (Waseem et al., 2007)	1			1						15	43
	c.121C > G	p.Leu41Val	reported as cause of disease, but no functional studies	Ellingford et al., 2016 (Ellingford et al., 2016a)	1				1						
	c.165G > A			de la Cerda et al., 2019 (de la Cerda et al., 2019)	1										
	c.172A > T	p.Lys58X		Zhang et al., 2016 (Zhang et al., 2016)	1										13
	c.196_197delAA	p.Lys66Aspfs*2		Xu et al., 2012 (Xu et al., 2012)	1		1	1							24
intron 2	c.177+1G > A		formerly IVS2+1 G > A	Sullivan et al., 2006a (Sullivan et al., 2006a)	1	1									
	c.177+1delG			Rivolta et al., 2006 (Rivolta et al., 2006)	1	1									
exon 3 c.178–238 (20aa)	c.217A > T	p.Lys73X		Eisenberger et al., 2013 (Eisenberger et al., 2013)	1			1							
	c.220C > T	p.Gln74X		Sullivan et al., 2006a (Sullivan et al., 2006a); Van Cauwenbergh et al., 2017 (Van Cauwenbergh et al., 2017); Kiser et al., 2019 (Kiser et al., 2019)	3			3						7	9
intron 3															
exon 4239-322	c.267delA	p.Glu89Aspfs*11		Sullivan et al., 2013 (Sullivan et al., 2013)	1		1								
		p.Leu107del4 ctGAGT		Aleman et al., 2009 (Aleman et al., 2009)	1		1								32
	c.319C > G			Rivolta et al., 2006 (Rivolta et al., 2006); Rio Frio et al., 2008 (Rio Frio et al., 2008b)	2	2									
intron 4	c.322 + 4_322+7del	p.?	2 families in MM paper, 3 families in Zhang paper	Zhang et al., 2016 (Zhang et al., 2016); Martin-Merida et al., 2018 (Martin-Merida et al., 2018)	5	5									19/27
	c.322+1G > A			Wu et al., 2018 (Wu et al., 2018); Kiser et al., 2019 (Kiser et al., 2019)	2	2									
	c.323-2A > G			Rivolta et al. (2006) (Rivolta et al., 2006)	1	1									
exon 5 c.323-421	c.328_330del	p.Ile110del	Reported as p.Ile109del in de Sousa Dias paper	de Sousa Dias et al., 2013 (de Sousa Dias et al., 2013); Martin-Merida et al., 2018 (Martin-Merida et al., 2018)	2					2					
	c.331_342del	p.His111_Ile114del	111 and 114 inc	Wang et al. (2003) (Wang et al., 2003)	1					1					
	c.341T > A	Ile114Asn		Wheway et al. (2019) (Wheway et al., 2019)	1				1						
	c.357_358delAA	p.Ser119Serfs*5	in 2 families and 1 sporadic case in Zheng paper	Xiao et al., 2017b (Xiao et al., 2017); Zheng et al., 2018 (Zheng et al., 2018)	2		2							6/10	
	c.358–359 del AA	p.Lys120Glufs*122		Gandra et al., 2008 (Gandra et al., 2008); Yang et al., 2015 (Yang et al., 2015)	2		2								
	c.359dupA			Hariri et al., 2018 (Hariri et al., 2018)	1										41
	c.359delA	p.Lys120Argfs*78		Carss et al., 2017 (Carss et al., 2017)	1		1								
	c.360dupA	p.K120fs*5		Glockle et al., 2014 (Glockle et al., 2014)	1		1								
	c.390delC	p.Asn131fs*67		Sullivan et al., 2006a (Sullivan et al., 2006a); Kiser et al., 2019 (Kiser et al., 2019)	2		2							10/10/17	16/21/48
	c.400delG	p.Asp134Ilefs		Ellingford et al., 2016 (Ellingford et al., 2016a)	1		1								
	c.413C > A	Thr138Lys		Waseem et al., 2007 (Waseem et al., 2007)	1				1					15,20	30
intron 5	c.421-2A > G			Jespersgaard et al., 2019 (Jespersgaard et al., 2019)	1	1									
	c.421-1G > A		formerly IVS5-1G > A	Xia et al., 2004 (Xia et al., 2004); Xi et al., 2005 (Xi et al., 2005)	2	2									
exon 6 c.421-527	c.421G > T	Glu141X		Sullivan et al., 2006a (Sullivan et al., 2006a)	1			1							
	c.433_434del	p.S145Pfs*8		Kurata et al., 2018 (Kurata et al., 2018)	1		1							7	7
	c.522_527 + 10del		Same family in these 2 papers	Ghazawy et al., 2007 (Ghazawy et al., 2007); Buskin et al., 2018 (Buskin et al., 2018)	1									30s	33
	c.525_526insAG			Kiser et al., 2019 (Kiser et al., 2019)	1		1							16	47
intron 6	c.527+1G > A		Described as p.IVS6+1G > T	Chakarova et al., 2006 (Chakarova et al., 2006); Martin-Merida et al., 2018 (Martin-Merida et al., 2018); Abdulridha-Aboud et al., 2016 (Abdulridha-Aboud et al., 2016)	3	3								13/48/21	13/48
	c.527+1G > T			Gandra et al., 2008 (Gandra et al., 2008); Kiser et al., 2019 (Kiser et al., 2019)	2	2									
	c.527+2T > G			Wu et al., 2018 (Wu et al., 2018)	1	1									
	c.527+2T > C			Audo et al., 2010 (Audo et al., 2010)	1	1									
	c.527+3A > G		In 2 families in Waseem paper. Reported as IVS 6 + 3 A > G in Ivings paper	Vithana et al., 2001 (Vithana et al., 2001); Waseem et al., 2007 (Waseem et al., 2007); Ivings et al., 2008 (Ivings et al., 2008); Ellingford et al., 2016 (Ellingford et al., 2016a); Xie et al., 2018 (Xie et al., 2018); Kiser et al., 2019 (Kiser et al., 2019)	7	7								20/29/28/27	52/30/70
	c.528–3_45del			Vithana et al., 2001 (Vithana et al., 2001); Sato et al., 2005 (Sato et al., 2005)	2	2									
	c.528–39_531del			Sullivan et al., 2013 (Sullivan et al., 2013)	1	1									
	c.528-1G > A			Waseem et al., 2007 (Waseem et al., 2007); Van Cauwenbergh et al., 2017 (Van Cauwenbergh et al., 2017)	2	2									20
exon 7 c.528-697	c.541G > T	p.Glu181X	2 families in MM paper	Pomares et al., 2010 (Pomares et al., 2010); Van Cauwenbergh et al., 2017 (Van Cauwenbergh et al., 2017); Martin-Merida et al., 2018 (Martin-Merida et al., 2018)	4			4							19, 23
	c.544_618del75bp	E182_E206del		Xu et al., 2012 (Xu et al., 2012)	1					1					24
	c.547delG	p.E183fs		Xiao et al., 2017 (Xiao et al., 2017)	1		1							5,6,7,8,10	
	c.548_580dup	p.Glu183_Met193dup		Tiwari et al., 2016 (Tiwari et al., 2016)	1						1			24	
	c.550_552del	p.Leu184del		Kiser et al., 2019 (Kiser et al., 2019)	1					1				71	71
	c.553G > T	p.Glu185X	de novo in Neveling paper	Neveling et al., 2012 (Neveling et al., 2012); van Huet et al., 2015 (van Huet et al., 2015)	2			1							
	c.562G > T	p.Glu188X		ClinVar (likely pathogenic)	1			1							
	c.580_581delGC	p.Leu195GlyFs		ClinVar (likely pathogenic)	1		1								
	c.580-581dup33bp			Vithana et al., 2001 (Vithana et al., 2001)	1						1				
	c.581C > A	Ala194Glu		Vithana et al., 2001 (Vithana et al., 2001)	1				1						
	c.590T > C	Leu197Pro		Bryant et al., 2019 (Bryant et al., 2019); Wu et al., 2018 (Wu et al., 2018)	2				2						
	c.615C > A	p.Tyr205X		ClinVar (pathogenic)	1			1							
	c.615C > G	p.Tyr205X		ClinVar (likely pathogenic)	1			1							
	c.615delC	p.Y205X		Xu et al., 2012 (Xu et al., 2012)	1			1							27
	c.616G > T	p.Glu206X		Wang et al., 2014 (Wang et al., 2014)	1			1							
	c.629delC			Huang et al., 2015 (Huang et al., 2015)	1		1								
	c.636delG	p.Met212fs*238		Sullivan et al., 2006a (Sullivan et al., 2006a); Bowne et al., 2011 (Bowne et al., 2011); Wang et al., 2014 (Wang et al., 2014)	3		3								
	c.646G > C	Ala216Pro		Vithana et al., 2001 (Vithana et al., 2001)	1				1						
	c.666_668del	p.Ile223del		Jespersgaard et al., 2019 (Jespersgaard et al., 2019)	1					1					
	c.673del	p.Ala225Hisfs*14		Jespersgaard et al., 2019 (Jespersgaard et al., 2019)	1		1								
intron 7	c.698-1G > A			Roberts et al., 2016 (Roberts et al., 2016); Birtel et al., 2018 (Birtel et al., 2018a)	2	2									
exon 8 c.698 - 855	c.709-734dup			Terray et al., 2017 (Terray et al., 2017)	1						1				
	732-737delins20bp	M244fsX248		Martinez-Gimeno et al., 2003 (Martinez-Gimeno et al., 2003)	1							1		6–20	
	c.736G > A	p.Ala246Thr		Xu et al., 2014 (Xu et al., 2012); Martin-Merida et al., 2018 (Martin-Merida et al., 2018)	2				2						
	c.741_742insA	p.Asn248Lysfs		ClinVar (likely pathogenic)	1		1								
	c.758_767del	p.Gly253fs*317		Sullivan et al., 2006a (Sullivan et al., 2006a); Kiser et al., 2019 (Kiser et al., 2019)	2		2							19	31
	c.763C > T	p.Gln255X		Wang et al., 2014 (Wang et al., 2014)	1			1							
	769-770insA	K257fs*277		Vithana et al., 2001 (Vithana et al., 2001); Martinez-Gimeno et al., 2003 (Martinez-Gimeno et al., 2003)	2		2							10–18	
	c.770dup	p.Thr258Aspfs*21		Vithana et al., 2001 (Vithana et al., 2001); Martin-Medira et al. (2018)	2		2								
	c.772_773del2insCAACATGCAACATCAT	p.(Thr258Glnfs)		Zhao et al., 2015 (Zhao et al., 2015)	1		1								
	c.781G > C	Gly261Arg		Xiao et al., 2017 (Xiao et al., 2017)	1				1						
	c.785delT	p.Phe262Serfs*59		Lim et al., 2009 (Lim et al., 2009)	1		1							<10	
	c.804delG	p.L268fs		Xiao et al., 2017 (Xiao et al., 2017)	1		1								
	c.808_809insC	p.His270Profs*8		Sullivan et al., 2013 (Sullivan et al., 2013)	1		1								
	c.815G > T	p.Gly272Val	predicted by Sullivan to be benign	Sullivan et al., 2006a (Sullivan et al., 2006a); Daiger et al., 2014 (Daiger et al., 2014)	2				2						
	c.816_830delCTACATCTACCACAG	p.Tyr273_Ser277del		Birtel et al., 2018 (Birtel et al., 2018b)	1					1					
	c.824_825insA	p.Y275X		Yang et al., 2013 (Yang et al., 2013)	1			1						12	
	c.828_829del	p.His276Glnfs*2		Martinez-Gimeno et al., 2003 (Martinez-Gimeno et al., 2003); Martin-Merida et al., 2018 (Martin-Merida et al., 2018)	2		2							5–20	
	c.838_841dupGTGC	p.Gln281Argfs*44		Carss et al., 2017 (Carss et al., 2017)	1		1								
	c.839T > G	p.Val280Gl		Birtel et al., 2018 (Birtel et al., 2018b)	1				1						
intron 8	c.855+1G > C			Lu et al., 2005 (Lu et al., 2005)	1	1									
	c.855+1G > T			Jespersgaard et al., 2019 (Jespersgaard et al., 2019)	1	1									
	c.855+1G > A			ClinVar (pathogenic)	1	1									
	c.856-2A > G			Rivolta et al., 2006 (Rivolta et al., 2006)	1	1									
exon 9 c.856 - 945	c.862C > T	p.Arg288Trp		Coussa et al., 2015 (Coussa et al., 2015)	1				1					66	68
	c.866_879delGGAAAGCGGCCCGG	p.R289Pfs*30		Villanueva et al., 2014 (Villanueva et al., 2014); Zhang et al., 2016 (Zhang et al., 2016)	2		2							2–16	7–63
	c.871G > C	Ala291Pro		Sullivan et al., 2006a (Sullivan et al., 2006a)	1				1						
	c.877_910del	p.Arg293_Arg304>Valfs*17		Rivolta et al., 2006 (Rivolta et al., 2006)	1		1								
	c.895T > C	Cys299Arg		Sullivan et al., 2006a (Sullivan et al., 2006a); Xu et al., 2012 (Xu et al., 2012); Martin-Merida et al., 2018 (Martin-Merida et al., 2018); Kiser et al., 2019 (Kiser et al., 2019)	4				4					21/27/41/63	27/44/63/65
	c.896G > A	p.Cys299Tyr		Bhatia et al., 2018 (Bhatia et al., 2018)	1				1						
	c.910C > T	p.Arg304Cys		Huang et al., 2015 (Huang et al., 2015); Hariri et al., 2018 (Hariri et al., 2018)	2				2						45
	c.914_915insTGT	p.Val305_Asp306insVal		Utz et al., 2013 (Utz et al., 2013)	1						1			30s	40s
	c.915_916insTGT	p.Val305_Asp306insCys		Sullivan et al., 2013 (Sullivan et al., 2013)	1						1				
	c.916G > A	p.Asp306Asn	reported as cause of disease, but functional studies not carried out	Ellingford et al., 2016 (Ellingford et al., 2016a)	1				1						
		p.307fs*15		Lu et al., 2013 (Lu et al., 2013)	1		1								
	c.939dup	p.Gly314Argfs*10		Fernandez-San Jose et al., 2015 (Fernandez-San Jose et al., 2015); Martin-Merida et al., 2018 (Martin-Merida et al., 2018)	2		2								
	c.940delG	p.Ala302Glnfs		ClinVar (pathogenic/likely pathogenic)	2		2								
intron 9	c.946–1 G > C			Bowne et al., 2011 (Bowne et al., 2011); Daiger et al. (2014) (Daiger et al., 2014)	2	2									
exon 10 c.946 - 1073	c.950delG	p.Gly316Alafs*4		ClinVar (pathogenic)	1		1								
	c.961A > T	p.Lys321X		Jespersgaard et al., 2019 (Jespersgaard et al., 2019)	1			1							
	c.967G > T	p.Glu323X		Ellingford et al., 2016 (Ellingford et al., 2016a)	1			1							
	c.973G > T	Glu325X		Sullivan et al., 2006a (Sullivan et al., 2006a)	1			1							
	c.978_982del	p.Lys327Argfs*146		Van Cauwenbergh et al., 2017 (Van Cauwenbergh et al., 2017)	1		1								
	c.992G > A	p.Trp331X		ClinVar (pathogenic)	1			1							
	c.994C > T	p.Gln332X		Ellingford et al., 2016 (Ellingford et al., 2016a)	1			1							
	c.997del	p.Glu333Serfs*5		Jespersgaard et al., 2019 (Jespersgaard et al., 2019)	1		1								
	c.1015C > T	p.Q339X		Xie et al., 2018 (Xie et al., 2018)	1			1							
	c.1035_1036insGC	p.Pro346Argfs*18		Wu et al., 2018 (Wu et al., 2018)	1		1								
	c.1048C > T	p.Gln350X		Eisenberger et al., 2013 (Eisenberger et al., 2013)	1			1							
	c.1060C > T	p.Arg354X		Sullivan et al., 2013 (Sullivan et al., 2013); Ellingford et al., 2016 (Ellingford et al., 2016a); Xiao et al., 2017 (Xiao et al., 2017); Wu et al., 2018 (Wu et al., 2018); Kurata et al., 2018 (Kurata et al., 2018); Kiser et al., 2019 (Kiser et al., 2019),	6			6						5/6/7/8/8/6/12	6/24
intron 10	c.1067_1073+8del			Eisenberger et al., 2013 (Eisenberger et al., 2013)	1	1									
	c.1073+1G > A			Sullivan et al., 2006a (Sullivan et al., 2006a); Kiser et al., 2019 (Kiser et al., 2019)	2	2								28/40	9/12/14/40
	c.1074-2 A > T	p.Tyr359Sfs*29		Yang et al., 2013 (Yang et al., 2013)	1	1								2–8	
	c.1074–1G > T	p.?		Martin-Merida et al., 2018 (Martin-Merida et al., 2018)	1	1									
exon 11 c.1074–1146 (24aa)	c.1077C > A	p.Tyr359X		Van Cauwenbergh et al., 2017 (Van Cauwenbergh et al., 2017)	1			1							
	c.1084delA	p.Met362X		Sullivan et al., 2013 (Sullivan et al., 2013); Kiser et al., 2019 (Kiser et al., 2019)	1			1						6	44
	c.1098delG	p.Leu366fs*1		Pan et al., 2014 (Pan et al., 2014)	1		1							5	22
	c.1115_1125del	p.Arg372Glnfs*99	Same family in Ivings and Buskin paper	Vithana et al., 2001 (Vithana et al., 2001); Ivings et al., 2008 (Ivings et al., 2008); Buskin et al., 2018 (Buskin et al., 2018)	2		2								Severe at 47
	c.1120C > T	p.Gln374X		Ellingford et al., 2016b (Ellingford et al., 2016b)	1			1							
	c.1129delC	p.Arg377Valfs*2		Carss et al., 2017 (Carss et al., 2017)	1		1								
	c.1142delG	p.Gly381fs*30	in more than 4 Japanese families in Koyanagi paper	Sato et al., 2005 (Sato et al., 2005); Taira et al., 2007 (Taira et al., 2007); Koyanagi et al., 2019 (Koyanagi et al., 2019)	6		6								30–45
intron 11	c.1146+2T > C	p.?		Waseem et al., 2007 (Waseem et al., 2007); Martin-Merida et al., 2018 (Martin-Merida et al., 2018)	2	2									18, 20
	c.1146+2T > A	p.?		Martin-Merida et al., 2018 (Martin-Merida et al., 2018)	1	1									
exon 12 c.1147 - 1275	c.1155-1159delGGACG/insAGGGATT	p.Asp386Glyfs*28		Sato et al., 2005 (Sato et al., 2005); Sullivan et al., 2006a (Sullivan et al., 2006a)	2		1							20	45
	c.1190dup	p.His398Profs*77		Jespersgaard et al., 2019 (Jespersgaard et al., 2019)	1		1								
	c.1205C > A	p.Ser402X		McLenachan et al., 2019 (McLenachan et al., 2019)	1			1							
	c.1215delG	p.G405fs*7		Dong et al., 2013 (Dong et al., 2013)	1		1							9	9,22,73
	c.1222C > T	p.Arg408Trp		Xiao et al., 2017 (Xiao et al., 2017)	1				1						
	c.1224dupG	p.Gln409Alafs*66		Wu et al., 2018 (Wu et al., 2018)	1		1								
	c.1226_1227insA	p.Thr410Aspfs*65		Xie et al., 2018 (Xie et al., 2018)	1										
	c.1234del	p.Val412X		Jespersgaard et al., 2019 (Jespersgaard et al., 2019)	1			1							
	c.1261_1262delTC	p.S421Qfs*53		Glockle et al., 2014 (Glockle et al., 2014)	1		1								
	c.1273C > T	p.Gln425X		ClinVar (pathogenic)	1			1							
intron 12															
exon 13 c.1276-1374	c.1291C > T	p.Gln431X		ClinVar (pathogenic)	1			1							
	c.1305T > A	p.Y435X		Huang et al., 2015 (Huang et al., 2015)	1			1							
	c.1373A > T	p.Gln458Leu		Xiao et al., 2017 (Xiao et al., 2017)	1				1						
intron 13	c.1374 + 654C > G		deep intronic	Rio Frio et al., 2009 (Rio Frio et al., 2009)	1	1									
exon 14 c.1375–1492 (39aa)	c.1462_1472del	p.Lys488Argfs*75		Martin-Merida et al., 2018 (Martin-Merida et al., 2018)	1		1								
Deletion	upstream			Jespersgaard et al., 2019 (Jespersgaard et al., 2019)	1								1		
Deletion	exons 1–14 (whole gene)			Ivings et al., 2008 (Ivings et al., 2008); Bowne et al., 2011 (Bowne et al., 2011); Eisenberger et al., 2013 (Eisenberger et al., 2013); Almoguera et al., 2015 (Almoguera et al., 2015) Hariri et al., 2018 (Hariri et al., 2018); Martin-Merida et al., 2018 (Martin-Merida et al., 2018)	6								6		18, severe at 65
30 kb deletion including putative promoter region of a novel gene OSCAR, the entire genomic content of genes NDUFA3, TFPT and most of the PRPF31 gene except for its terminal exon exon 14.				Abu-Safieh et al., 2006 (Abu-Safieh et al., 2006)	1								1	6–30	
112 kb deletion encompassing over 90% of PRPF31 and five upstream genes: TFPT, OSCAR, NDUFA3, TARM-1, and VSTM-1				Rose et al., 2011 (Rose et al., 2011)	1								1		
58.7 kb deletion including TFPT, NDUFA3, OSCAR genes and 11 exons of the PRPF31		2 families in Sweden		Kohn et al., 2009 (Kohn et al., 2009); Golovleva et al., 2010 (Golovleva et al., 2010)	2								2		50s
Deletion	exon 1			Martin-Merida et al., 2018 (Martin-Merida et al., 2018)	1								1		
Deletion	exons 1–3			Birtel et al., 2018 (Birtel et al., 2018a)	1								1		
12 kb deletion including exons 1–3 of PRPF31				Dong et al., 2013 (Dong et al., 2013)	1								1		
Deletion	exons 1–5			Eisenberger et al., 2013 (Eisenberger et al., 2013); Birtel et al., 2018 (Birtel et al., 2018a)	2								2		
Deletion	Intron 1			Jespersgaard et al., 2019 (Jespersgaard et al., 2019)	1								1		
Deletion	exons 2-3			Jespersgaard et al., 2019 (Jespersgaard et al., 2019)	1								1		
Duplication	exons 2-5			Martin-Merida et al., 2018 (Martin-Merida et al., 2018)	1								1		
Deletion	exons 2-5			Jespersgaard et al., 2019 (Jespersgaard et al., 2019)	1								1		
Deletion	exons 2-14			Jespersgaard et al., 2019 (Jespersgaard et al., 2019)	1								1		
Duplication	exons 4-5			Jespersgaard et al., 2019 (Jespersgaard et al., 2019)	1								1		
Deletion	exons 4-13			Weisschuh et al., 2016 (Weisschuh et al., 2016)	1								1		
Deletion	exon 9			Martin-Merida et al., 2018 (Martin-Merida et al., 2018)	1								1		
Promoter mutation				Rose et al., 2012 (Rose et al., 2012)	1								1		
Insertion/deletion	149 bp deleted/640 bp inserted	hg17 Deletion of 59,310,880–59,311,028/insertion of 59,292,594–59,291,955 reverse comp.		Sullivan et al., 2006b (Sullivan et al., 2006b)	1								1	14/16/25/46	37/46/50/52/77
Deletion	4.8 kb	hg 17 59,315,842–59,320,684		Sullivan et al., 2006b (Sullivan et al., 2006b)	1								1		
Deletion	11.3 kb	hg17 59,314,340–59,325,633		Sullivan et al., 2006b (Sullivan et al., 2006b)	1								1		
Deletion	32–42 kb	hg17 5′ breakpoint: 59,290,949–59,295,848; ′ breakpoint: 59,328,550–59,333,288		Sullivan et al., 2006b (Sullivan et al., 2006b)	1								1		
Deletion	>44.8 kb	hg17 5′ breakpoint: <59,283,753; 3′ breakpoint: 59,328,550–59,333,288		Sullivan et al., 2006b (Sullivan et al., 2006b)	1								1		
Deletion	19:54622548–54633842del11295			Carss et al., 2017 (Carss et al., 2017)	1								1		
Deletion	19:54632279–54632481del203			Carss et al., 2017 (Carss et al., 2017)	1								1

The majority of reported mutations in *PRPF31* are presumed loss-of-function variants including frameshift (51 different variants reported in 70 different families), splice site (30 variants in 52 families), nonsense (30 variants in 40 families) or large-scale insertions or deletions (25 variants in 32 families), which are predicted to lead to complete loss of expression of protein from the affected allele. *PRPF31* is highly intolerant to loss-of-function with a probability of being loss-of-function intolerant (pLI) score of 0.98 (Lek et al., 2016). A pLI score of >0.9 indicates that a gene is intolerant of protein-truncating variation (Lek et al., 2016) and thus loss-of-function variants in *PRPF31* are highly likely to cause disease through a haploinsufficiency disease mechanism (discussed in more detail later). However, it is important to note that whilst frameshift, consensus splice site, nonsense and large indel variants are often assumed to cause loss-of-function, this is not always the case, particularly when frameshift or nonsense variants are found in the final exon or C-terminal portion of the penultimate exon; transcripts from genes with such variants are likely to evade nonsense mediated decay (Ziegler et al., 2019). At least 3 frameshift or nonsense mutations in the final two exons of *PRPF31* have been reported as pathogenic, but functional study is required to confirm pathogenicity (Martin-Merida et al., 2018; Huang et al., 2015). Similarly, consensus splice site mutations are often also assumed to cause complete loss of wild-type protein expression from the affected allele, when in fact the complex mechanisms of alternative splicing may lead to production of a truncated protein, particularly if the splicing change produces an in-frame transcript. In several cases where mutations are assumed to be causing loss-of-function through haploinsufficiency, in addition to presumed loss-of-function variants, at least 19 missense variants have been reported in *PRPF31* as being pathogenic. Gene constraint metrics, which provide quantitative measures of the extent to which a gene can tolerate change, indicate that *PRPF31* gene is highly intolerant to missense variants (Z = 3.27) (Samocha et al., 2014; Lek et al., 2016). Missense mutations in *PRPF31* tend to reduce the solubility of protein so it does not translocate into nucleus efficiently after being translated in the cytoplasm (Deery et al., 2002; Bryant et al., 2019; Wheway et al., 2019), effectively leading to a loss of this protein. However, only 4 missense variants have been functionally studied *in vitro*, and a comprehensive study of reported missense variants is required to confirm the functional effect of pathogenic variants, and indeed the pathogenicity of reported variants. At least one variant originally described as a missense variant was later confirmed to be affecting splicing (Rio Frio et al., 2008b) and it is possible that other variants classified as missense, both recognised pathogenic and those currently considered non-pathogenic, may in fact be impacting upon splicing of *PRPF31*. Furthermore, non-synonymous rare variants may impact on splicing. It is therefore likely that the rate of pathogenic variants affecting splicing in *PRPF31* is underestimated.

### 1.6 Genotype-phenotype correlation

**Fig. 4:**
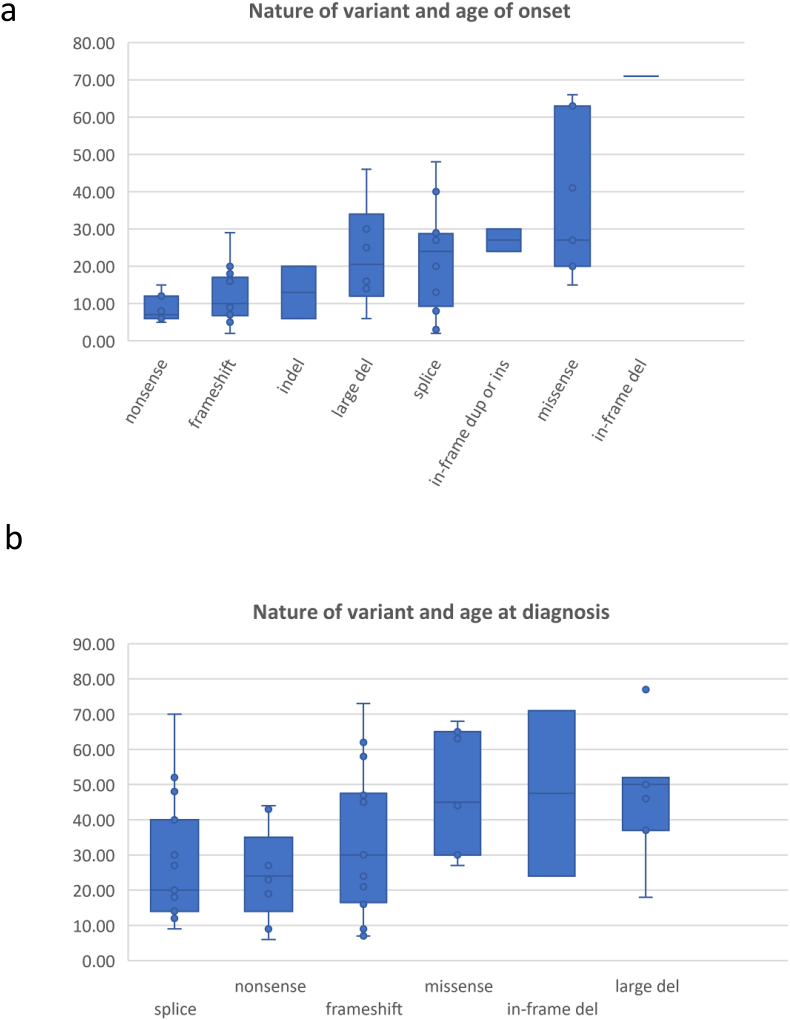
(a) Box and whisker plots showing upper and lower limits, median and interquartile range of reported age of onset of RP patients with different types of variant in *PRPF31* (b) Box and whisker plots showing upper and lower limits, median and interquartile range of reported age of diagnosis of RP patients with different types of variant in *PRPF31*.

We reviewed the literature and recorded the age of onset of first symptoms, and age of diagnosis, where it was reported alongside specific genetic variants. Age of onset of first symptoms (usually night-blindness) is lowest in patients with nonsense, frameshift or indel variants, with median age of onset between 8 and 12 years of age. Patients with large deletions or splice variants tend to show first symptoms at a slightly later median age of 20–24. Patients with in-frame duplications, insertions or missense variants show the latest median age of onset of first symptoms, around 27 years of age (Fig. 4a). The difference in age of onset between the different types of mutation is statistically significant (one-way ANOVA p = 5.76 × 10^−5^).

We also recorded the age of diagnosis where it was reported alongside specific *PRPF31* genetic variants. In this case, patients with nonsense, frameshift or splice variants were diagnosed at a median age of 20–30 years (usually because of loss of peripheral vision alongside night blindness), whereas patients with missense variants, in-frame deletions or large deletions tended to be diagnosed between the ages of 45 and 50 (Fig. 4b). The difference in age of diagnosis between the different types of mutation is statistically significant (one-way ANOVA p = 0.030).

There is no significant correlation between location of the variant in the gene and age of onset of symptoms or age of diagnosis.

It is an interesting observation, made in several studies and confirmed here, that patients with large-scale deletions, including multi-exon and whole gene deletions have the latest age of diagnosis. There is a clear difference in age of diagnosis of patients with large-scale deletions compared to patients with nonsense mutations or splice mutations although this is not statistically significant after correction for multiple testing (two-tailed unpaired *t*-test p = 0.016 and p = 0.032 respectively, p = 0.24 and p = 0.48 respectively after Bonferroni correction) (Fig. 4b). It could be postulated that there is an element of dominant negative effect at play in cases of nonsense, frameshift, indel, in-frame and missense variants compared to large deletions. This is a feature of the disease which should be considered when designing targeted therapies. The abundance of loss-of-function mutations, including complete gene deletions, in *PRPF31* patients has led to a consensus view that haploinsufficiency is the disease mechanism in this form of RP(Abu-Safieh et al., 2006; Rio Frio et al., 2008b; Rose and Bhattacharya, 2016). This has influenced approaches for targeted therapies, namely gene augmentation approaches, which involve replacing a wild-type copy of the coding sequence of PRPF31 into the subretinal space of patients. This may not be fully effective in patients with genetic variants which have a dominant negative effect as well as a haploinsufficiency effect, and as a result other approaches for treatment may need to be investigated. These findings are supported by other recent work which also proposes a combined haploinsufficiency and dominant-negative disease mechanism in disease associated *with PRPF31* mutations(Valdés-Sánchez et al., 2019). Study of the *Prpf31*^*p.A216P/+*^ mouse has shown that heterozygous missense mutations in *Prpf31* lead to aggregation of both wild-type and mutant protein in the cytoplasm of the RPE cells of mice, leading to overexpression of HSP70 family proteins(Valdés-Sánchez et al., 2019). This work suggests that over-expression of these HSP70 proteins may be a target for therapy in PRPF31 patients, rather than targeting PRPF31 itself(Valdés-Sánchez et al., 2019).

## 2 Opportunities for therapies

### 2.1 Gene augmentation therapy

As a result of the abundance of loss-of-function variants in *PRPF31* gene augmentation has been postulated as a potential therapeutic approach to treat this form of RP(Hafler et al., 2016). The coding sequence of *PRPF31* is only 1.5 kb, well within the limits of current gene therapy vectors, and a *PRPF31* heterozygous knockout mouse is available for study, although it only develops very late onset retinal degeneration which may be more characteristic of age-related macular degeneration than RP(Farkas et al., 2014). Researchers have begun preparatory work to define pre-treatment characteristics of RP associated with *PRPF31* mutations in order to be able to assess the effectiveness of AAV-mediated *PRPF31* gene augmentation therapy(Hafler et al., 2016). These researchers have also patented *PRPF31* gene therapy by AAV2 delivery (International Publication Number WO2016144892A1) and, shown rescue of key cellular disease phenotypes including phagocytosis, ciliogenesis, cell morphology and barrier function in mutant *PRPF31*^*+/−*^ RPE derived from patient iPSCs after deliver of PRPF31(Brydon et al., 2019).

### 2.2 Antisense oligonucleotide therapy

If the majority of genetic variants have some dominant negative effect, it is important to consider other potential therapeutic approaches. These include antisense oligonucleotides (ASOs) which can bind pre-mRNA or mRNA and modulate splicing of PRPF31 pre-mRNA or inhibit translation of the mRNA. In addition, siRNAs, shRNAs or gapmer-style ASOs can be used to completely silence a gene, which when combined with gene augmentation could potentially correct a disease with dominant negative effects. This approach has been successfully applied to the treatment of RP associated with dominant negative RHO mutations(Cideciyan et al., 2018). Splice-switching ASOs can be used to bind and mask deep intronic variants which introduce novel splice sites (such as the deep intronic variant in intron 13 reported in Rio Frio et al. (2009)(Rio Frio et al., 2009). Alternatively, they can be used to induce exon skipping of an in-frame exon (ie an exon with a multiple of 3 base pairs) carrying a frameshift or null variant, in order to remove this variant and restore the reading frame. Three of the fourteen exons in PRPF31 have multiples of 3 base pairs; exons 3, 11 and 14 (Fig. 2, Fig. 3). These are also relatively small exons, and do not encode functional important domains of the protein (Fig. 2) so they could be targeted for skipping without removing large or functionally important regions of the protein. This could have the effect of reverting a severe, early-onset frameshift or nonsense variant into a less severe splice or in-frame deletion variant, although the exon skipping would affect both alleles, mutant and normal, so the effect may be like having a homozygous exon deletion. According to the genotype-phenotype data in this study, this could delay age of onset from 8 to 10 years of age to 25 years of age or later. If this exon skipping approach led to a disease more like in-frame deletions, this could delay age of diagnosis (taken as a proxy for loss of peripheral vision) from 25 to 30 years of age to 47 years of age. This could potentially preserve vision in the working age of these individuals. This is a promising approach in theory, and such drugs are already being developed for a range of previously untreatable genetic conditions.(Scoles and Pulst, 2019; Levin, 2019; Khan et al., 2019). A clinically available splice-switching ASO drug (nusinersen) based on 2′O-methoxyethyl phosphorothioate chemistry has been successfully developed for the treatment of the neurodegenerative disease spinal muscular atrophy (approved by NICE) and a similar type of drug (eteplirsen) utilising phosphorodiamidate morpholino chemistry has been developed for treatment of certain forms of Duchenne muscular dystrophy(Finkel et al., 2017; Mendell et al., 2016). Intraocularly delivered ASO drugs are also currently undergoing clinical trials for a specific form of Leber congenital amaurosis caused by a *CEP290* deep intronic mutation (ClinicalTrials.gov NCT03140969). ASOs are highly versatile drugs, being sequence-specific in their action, titratable in dosage, and in the setting of a well-defined and enclosed target organ such as the eye, straightforward to deliver by direct intravitreal or subretinal injection. However, there are limited numbers of affected individuals who could be treated by targeting these regions of *PRPF31* (around 27 families).

### 2.3 Gene independent approaches

As RP associated with *PRPF31* is so genetically diverse, (172 different reported variants in 240 different families or simplex cases) gene independent approaches are extremely attractive alternatives to gene therapies. These include stem cell therapies and bionic retinal implants. Stem cell therapies are both gene and disease-agnostic, and can replace lost retinal cells, whereas gene therapies can only recover function of intact cells. Stem cell therapies are closest to being effective in replacement of the retinal pigment epithelium (RPE), which has no neural connection. It is more challenging to regenerate functional neural retina. Recent studies have shown promising results in stem cell replacement of RPE for treatment of age-related macular degeneration(da Cruz et al., 2018; Kashani et al., 2018). Bioinic retinae such as the Argus II(Finn et al., 2018) are able to restore limited light and shape perception in people with end-stage retinal disease and limited to no remaining retinal function.

## 3 Conclusions and future perspective

Gene therapy offers real potential for treatment of a range of currently untreatable inherited retinal degenerations. As the second most common cause of adRP, and a relatively small gene, *PRPF31* is becoming a focus for gene augmentation therapy(Brydon et al., 2019). This approach assumes a disease mechanism of haploinsufficiency, of which there is considerable evidence. However, new data presented here supports the recently proposed theory that, except in cases of complete exon or gene deletion, dominant negative effects may contribute to disease progression in RP associated with *PRPF31* variants (Valdés-Sánchez et al., 2019), and that gene augmentation therapy may not be as effective in patients with missense, nonsense or splice mutations compared to whole exon or whole gene deletions. Whilst it is important to pursue these studies, data from knockout mice must be interpreted with caution when translating into human studies, and alternatively approaches must also be investigated. These include antisense oligonucleotide therapy targeting suitable exons, and gene-independent approaches. With several potential therapeutic approaches under investigation, there is real hope that treatment options for this disorder will be available to the next generation of patients.

## Author contributions

GW undertook the literature review, collected and tabulated genotype and phenotype data and prepared figures. EG performed statistical analysis of data. GW, AD and DB wrote the manuscript.
